# 2-(4,6-Dimethyl­pyrimidin-2-ylsulfan­yl)-*N*-phenyl­acetamide

**DOI:** 10.1107/S1600536808007940

**Published:** 2008-03-29

**Authors:** Li-Xin Gao, Guang-Jun Fang, Jin-Guo Feng, Dong Liang, Wei Wang

**Affiliations:** a School of Chemical Engineering, University of Science and Technology Liaoning, Anshan 114002, People’s Republic of China

## Abstract

In the title compound, C_14_H_15_N_3_OS, the phenyl ring is almost perpendicular to the dimethyl­pyrimidine group, with a dihedral angle of 88.1 (3)°. The C*sp* ^2^—S bond of 1.759 (3) Å is significantly shorter than the C*sp* ^3^—S bond of 1.795 (3) Å due to the *p*–π conjugation.

## Related literature

For related literature, see: Koike *et al.* (1999 ▶); Liang *et al.* (2008 ▶); Wang *et al.* (2004 ▶, 2005 ▶).
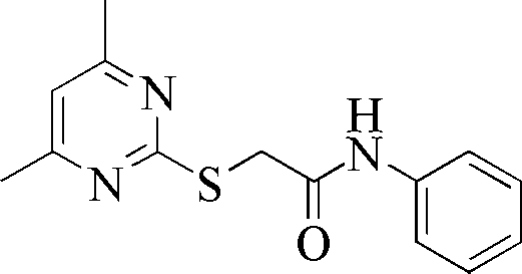


## Experimental

### 

#### Crystal data

C_14_H_15_N_3_OS
*M* *_r_* = 273.35
Orthorhombic, 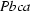

*a* = 9.1691 (17) Å
*b* = 15.485 (3) Å
*c* = 20.798 (4) Å
*V* = 2952.9 (9) Å^3^
*Z* = 8
Mo *K*α radiation
μ = 0.21 mm^−1^
*T* = 571 (2) K
0.28 × 0.24 × 0.14 mm

#### Data collection

Bruker SMART CCD area-detector diffractometer
Absorption correction: multi-scan (*SADABS*; Sheldrick, 1996 ▶) *T*_min_ = 0.942, *T*_max_ = 0.971
14235 measured reflections
2606 independent reflections
1494 reflections with *I* > 2σ(*I*)
*R*_int_ = 0.073

#### Refinement

*R*[*F* ^2^ > 2σ(*F* ^2^)] = 0.040
*wR*(*F* ^2^) = 0.116
*S* = 1.01
2606 reflections
175 parameters
H-atom parameters constrained
Δρ_max_ = 0.17 e Å^−3^
Δρ_min_ = −0.16 e Å^−3^

### 

Data collection: *SMART* (Bruker, 1997 ▶); cell refinement: *SMART*; data reduction: *SAINT* (Bruker, 1997 ▶); program(s) used to solve structure: *SHELXS97* (Sheldrick, 2008 ▶); program(s) used to refine structure: *SHELXL97* (Sheldrick, 2008 ▶); molecular graphics: *SHELXTL* (Sheldrick, 2008 ▶); software used to prepare material for publication: *SHELXTL*.

## Supplementary Material

Crystal structure: contains datablocks global, I. DOI: 10.1107/S1600536808007940/at2550sup1.cif

Structure factors: contains datablocks I. DOI: 10.1107/S1600536808007940/at2550Isup2.hkl

Additional supplementary materials:  crystallographic information; 3D view; checkCIF report

## Figures and Tables

**Fig. 1.**
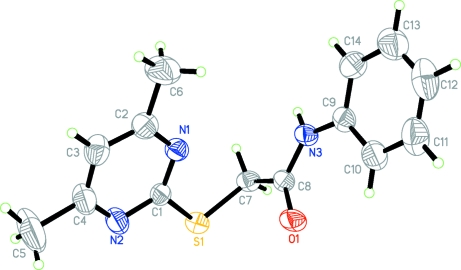
View of the molecule of (I) showing the atom-labelling scheme. Displacement ellipsoids are drawn at the 30% probability level.

## supplementary crystallographic information

### Comment

Acetamide is an important class of medical intermidate. Many biologically active compounds are synthesized by using acetamide (Koike *et al.*, 1999). We have reported the synthesis and crystal structure of a acetamide compound, 1,1'-diphenyl-5,5'-[*o*-phenylenebis(methylenethio)]di-1*H*-tetrazole (Liang *et al.*, 2008). Now, a new acetamide derivative, namely 2-(4,6-dimethylpyrimidin-2- ylsulfanyl)-*N*-phenylacetamide (I) are prepared from the reaction of 2-thio-4,6- dimethylpyrimidine with 2-chloro-*N*-phenylacetamide. We present its crystal structure here.

The title compound contains a benzene ring and a dimethylpyrimidine ring. The two methyl groups attached to the pyrimidine ring don't deviate from the pyrimidine ring, with an r.m.s. of 0.0082 (4) Å. The dihedral angle between the benzene ring and dimethylpyrimidine ring is 91.9 (3)°, which indicates that the two aromatic rings are almost perpendicular. The O1—C8—N3—C9 and C8—N3—C9—C14 torsion angles are 2.5 (4) and 164.3 (3)°, indicating that the acetamide is planar with the benzene ring.

Due to the *p*-π conjugation, the C*sp*^2^—S bond [S1—C1 = 1.759 (3) Å] is significantly shorter than the C*sp*^3^—S bond [C7—S1 = 1.795 (3) Å]. These values compare with the values reported in the literatures (Wang *et al.*, 2004, 2005).

### Experimental

The title compound was synthesized by the reaction of 2-thio-4,6-dimethyl- pyrimidine(2 mmol) with 2-chloro-*N*-phenylacetamide (2 mmol) in refluxing ethanol (40 ml). Single crystals suitable for X-ray analysis were grown by slow evaporation of a chloroform-acetone (1:5 *v*/*v*) solution.

### Refinement

All H atoms were positioned geometrically and refined as riding [N—H = 0.86Å and C—H = 0.93–0.97 Å]. For the NH, CH and CH_2_ groups, *U*_iso_(H) values were set equal to 1.2*U*_eq_(C) and for the methyl groups they were set equal to 1.5*U*_eq_(C).

### Crystal data

**Table:**

C_14_H_15_N_3_OS	*F*_000_ = 1152
*M**_r_* = 273.35	*D*_x_ = 1.230 Mg m^−^^3^
Orthorhombic, *P**b**c**a*	Mo *K*α radiation λ = 0.71073 Å
Hall symbol: -P 2ac 2ab	Cell parameters from 2395 reflections
*a* = 9.1691 (17) Å	θ = 2.8–22.1º
*b* = 15.485 (3) Å	µ = 0.22 mm^−^^1^
*c* = 20.798 (4) Å	*T* = 571 (2) K
*V* = 2952.9 (9) Å^3^	Block, colourless
*Z* = 8	0.28 × 0.24 × 0.14 mm

### Data collection

**Table:**

Bruker SMART CCD area-detector diffractometer	2606 independent reflections
Radiation source: fine-focus sealed tube	1494 reflections with *I* > 2σ(*I*)
Monochromator: graphite	*R*_int_ = 0.073
*T* = 571(2) K	θ_max_ = 25.0º
φ and ω scans	θ_min_ = 2.0º
Absorption correction: multi-scan(SADABS; Sheldrick, 1996)	*h* = −8→10
*T*_min_ = 0.942, *T*_max_ = 0.971	*k* = −18→18
14235 measured reflections	*l* = −22→24

### Refinement

**Table:**

Refinement on *F*^2^	Hydrogen site location: inferred from neighbouring sites
Least-squares matrix: full	H-atom parameters constrained
*R*[*F*^2^ > 2σ(*F*^2^)] = 0.040	*w* = 1/[σ^2^(*F*_o_^2^) + (0.044*P*)^2^ + 0.9487*P*] where *P* = (*F*_o_^2^ + 2*F*_c_^2^)/3
*wR*(*F*^2^) = 0.116	(Δ/σ)_max_ = 0.001
*S* = 1.01	Δρ_max_ = 0.17 e Å^−^^3^
2606 reflections	Δρ_min_ = −0.16 e Å^−^^3^
175 parameters	Extinction correction: SHELXL, Fc^*^=kFc[1+0.001xFc^2^λ^3^/sin(2θ)]^-1/4^
Primary atom site location: structure-invariant direct methods	Extinction coefficient: 0.0155 (11)
Secondary atom site location: difference Fourier map	

### Special details

**Table:**

Geometry. All e.s.d.'s (except the e.s.d. in the dihedral angle between two l.s. planes) are estimated using the full covariance matrix. The cell e.s.d.'s are taken into account individually in the estimation of e.s.d.'s in distances, angles and torsion angles; correlations between e.s.d.'s in cell parameters are only used when they are defined by crystal symmetry. An approximate (isotropic) treatment of cell e.s.d.'s is used for estimating e.s.d.'s involving l.s. planes.
Refinement. Refinement of *F*^2^ against ALL reflections. The weighted *R*-factor *wR* and goodness of fit *S* are based on *F*^2^, conventional *R*-factors *R* are based on *F*, with *F* set to zero for negative *F*^2^. The threshold expression of *F*^2^ > σ(*F*^2^) is used only for calculating *R*-factors(gt) *etc*. and is not relevant to the choice of reflections for refinement. *R*-factors based on *F*^2^ are statistically about twice as large as those based on *F*, and *R*- factors based on ALL data will be even larger.

### Fractional atomic coordinates and isotropic or equivalent isotropic displacement parameters (Å^2^)

**Table:**

	*x*	*y*	*z*	*U*_iso_*/*U*_eq_	
S1	0.10446 (8)	0.82693 (5)	0.31856 (3)	0.0566 (3)	
O1	0.08455 (18)	0.69624 (13)	0.20969 (9)	0.0658 (6)	
N1	0.1800 (2)	0.67674 (14)	0.37036 (10)	0.0540 (6)	
C8	0.2078 (3)	0.70685 (17)	0.23078 (12)	0.0498 (7)	
C1	0.0880 (3)	0.74194 (16)	0.37418 (12)	0.0480 (7)	
N3	0.3186 (2)	0.65184 (14)	0.22060 (10)	0.0539 (6)	
H3A	0.4015	0.6669	0.2364	0.065*	
C7	0.2494 (3)	0.78653 (16)	0.26898 (13)	0.0541 (7)	
H7A	0.2793	0.8316	0.2394	0.065*	
H7B	0.3324	0.7728	0.2961	0.065*	
C9	0.3164 (3)	0.57251 (18)	0.18732 (13)	0.0538 (7)	
N2	−0.0195 (3)	0.75292 (15)	0.41631 (11)	0.0665 (7)	
C2	0.1643 (3)	0.61439 (19)	0.41447 (15)	0.0643 (8)	
C14	0.4321 (4)	0.51737 (19)	0.19623 (14)	0.0703 (9)	
H14	0.5079	0.5332	0.2235	0.084*	
C10	0.2056 (3)	0.5472 (2)	0.14672 (15)	0.0779 (10)	
H10	0.1266	0.5837	0.1396	0.093*	
C3	0.0557 (4)	0.6186 (2)	0.45961 (14)	0.0707 (9)	
H3	0.0440	0.5748	0.4898	0.085*	
C4	−0.0353 (4)	0.6890 (2)	0.45926 (15)	0.0774 (10)	
C13	0.4371 (5)	0.4388 (2)	0.16514 (18)	0.0922 (12)	
H13	0.5167	0.4025	0.1712	0.111*	
C12	0.3259 (6)	0.4141 (2)	0.12546 (18)	0.0939 (12)	
H12	0.3289	0.3608	0.1048	0.113*	
C6	0.2714 (4)	0.5413 (2)	0.41157 (19)	0.1032 (13)	
H6A	0.2749	0.5188	0.3686	0.155*	
H6B	0.2415	0.4965	0.4406	0.155*	
H6C	0.3663	0.5618	0.4236	0.155*	
C11	0.2113 (5)	0.4679 (3)	0.11651 (17)	0.0930 (12)	
H11	0.1354	0.4512	0.0896	0.112*	
C5	−0.1579 (6)	0.6993 (3)	0.5073 (2)	0.147 (2)	
H5A	−0.1525	0.7555	0.5266	0.221*	
H5B	−0.1491	0.6559	0.5400	0.221*	
H5C	−0.2498	0.6929	0.4857	0.221*	

### Atomic displacement parameters (Å^2^)

**Table:**

	*U*^11^	*U*^22^	*U*^33^	*U*^12^	*U*^13^	*U*^23^
S1	0.0512 (4)	0.0549 (4)	0.0636 (5)	0.0038 (4)	0.0011 (4)	0.0005 (4)
O1	0.0307 (10)	0.0895 (15)	0.0773 (13)	−0.0027 (10)	−0.0043 (9)	−0.0168 (11)
N1	0.0526 (14)	0.0541 (13)	0.0554 (14)	0.0043 (12)	−0.0011 (11)	−0.0041 (12)
C8	0.0318 (15)	0.0690 (17)	0.0486 (16)	−0.0061 (14)	0.0067 (12)	0.0041 (14)
C1	0.0472 (16)	0.0529 (16)	0.0438 (15)	−0.0019 (14)	−0.0039 (13)	−0.0085 (12)
N3	0.0309 (12)	0.0714 (16)	0.0595 (14)	−0.0028 (11)	0.0009 (10)	−0.0078 (12)
C7	0.0362 (14)	0.0618 (16)	0.0641 (18)	−0.0076 (13)	−0.0001 (13)	0.0049 (14)
C9	0.0445 (16)	0.0656 (18)	0.0514 (17)	−0.0071 (15)	0.0112 (14)	0.0026 (15)
N2	0.0706 (17)	0.0670 (16)	0.0620 (16)	0.0121 (14)	0.0178 (14)	0.0002 (13)
C2	0.072 (2)	0.0547 (18)	0.066 (2)	0.0035 (16)	−0.0021 (18)	−0.0043 (16)
C14	0.075 (2)	0.069 (2)	0.067 (2)	−0.0001 (18)	−0.0013 (17)	0.0073 (16)
C10	0.059 (2)	0.101 (3)	0.073 (2)	−0.0015 (18)	0.0026 (17)	−0.026 (2)
C3	0.092 (2)	0.0622 (19)	0.0583 (19)	−0.0038 (19)	0.0064 (18)	0.0034 (15)
C4	0.089 (2)	0.078 (2)	0.065 (2)	0.009 (2)	0.0241 (19)	0.0003 (18)
C13	0.124 (4)	0.066 (2)	0.087 (3)	0.019 (2)	0.012 (2)	0.007 (2)
C12	0.137 (4)	0.071 (2)	0.074 (3)	−0.017 (3)	0.028 (3)	−0.013 (2)
C6	0.113 (3)	0.075 (2)	0.122 (3)	0.031 (2)	0.010 (3)	0.016 (2)
C11	0.091 (3)	0.109 (3)	0.079 (3)	−0.018 (3)	0.010 (2)	−0.030 (2)
C5	0.175 (5)	0.138 (4)	0.129 (4)	0.045 (3)	0.100 (3)	0.029 (3)

### Geometric parameters (Å, °)

**Table:**

S1—C1	1.759 (3)	C14—H14	0.9300
S1—C7	1.795 (3)	C10—C11	1.380 (4)
O1—C8	1.223 (3)	C10—H10	0.9300
N1—C1	1.318 (3)	C3—C4	1.373 (4)
N1—C2	1.340 (3)	C3—H3	0.9300
C8—N3	1.343 (3)	C4—C5	1.512 (5)
C8—C7	1.516 (3)	C13—C12	1.367 (5)
C1—N2	1.330 (3)	C13—H13	0.9300
N3—C9	1.410 (3)	C12—C11	1.354 (5)
N3—H3A	0.8600	C12—H12	0.9300
C7—H7A	0.9700	C6—H6A	0.9600
C7—H7B	0.9700	C6—H6B	0.9600
C9—C14	1.375 (4)	C6—H6C	0.9600
C9—C10	1.378 (4)	C11—H11	0.9300
N2—C4	1.341 (4)	C5—H5A	0.9600
C2—C3	1.370 (4)	C5—H5B	0.9600
C2—C6	1.500 (4)	C5—H5C	0.9600
C14—C13	1.378 (4)		
			
C1—S1—C7	100.40 (13)	C11—C10—H10	119.9
C1—N1—C2	116.2 (2)	C2—C3—C4	118.4 (3)
O1—C8—N3	123.9 (2)	C2—C3—H3	120.8
O1—C8—C7	122.0 (2)	C4—C3—H3	120.8
N3—C8—C7	114.1 (2)	N2—C4—C3	121.6 (3)
N1—C1—N2	127.7 (2)	N2—C4—C5	116.3 (3)
N1—C1—S1	118.6 (2)	C3—C4—C5	122.1 (3)
N2—C1—S1	113.7 (2)	C12—C13—C14	120.4 (4)
C8—N3—C9	128.2 (2)	C12—C13—H13	119.8
C8—N3—H3A	115.9	C14—C13—H13	119.8
C9—N3—H3A	115.9	C11—C12—C13	119.3 (3)
C8—C7—S1	113.47 (17)	C11—C12—H12	120.4
C8—C7—H7A	108.9	C13—C12—H12	120.4
S1—C7—H7A	108.9	C2—C6—H6A	109.5
C8—C7—H7B	108.9	C2—C6—H6B	109.5
S1—C7—H7B	108.9	H6A—C6—H6B	109.5
H7A—C7—H7B	107.7	C2—C6—H6C	109.5
C14—C9—C10	118.4 (3)	H6A—C6—H6C	109.5
C14—C9—N3	117.6 (3)	H6B—C6—H6C	109.5
C10—C9—N3	124.0 (3)	C12—C11—C10	121.0 (4)
C1—N2—C4	115.1 (2)	C12—C11—H11	119.5
N1—C2—C3	120.9 (3)	C10—C11—H11	119.5
N1—C2—C6	116.5 (3)	C4—C5—H5A	109.5
C3—C2—C6	122.6 (3)	C4—C5—H5B	109.5
C9—C14—C13	120.7 (3)	H5A—C5—H5B	109.5
C9—C14—H14	119.6	C4—C5—H5C	109.5
C13—C14—H14	119.6	H5A—C5—H5C	109.5
C9—C10—C11	120.3 (3)	H5B—C5—H5C	109.5
C9—C10—H10	119.9		
			
C2—N1—C1—N2	−0.7 (4)	C10—C9—C14—C13	0.1 (4)
C2—N1—C1—S1	178.98 (19)	N3—C9—C14—C13	−179.9 (3)
C7—S1—C1—N1	3.4 (2)	C14—C9—C10—C11	0.6 (5)
C7—S1—C1—N2	−176.94 (19)	N3—C9—C10—C11	−179.3 (3)
O1—C8—N3—C9	−2.5 (4)	N1—C2—C3—C4	−1.0 (4)
C7—C8—N3—C9	179.8 (2)	C6—C2—C3—C4	178.7 (3)
O1—C8—C7—S1	34.4 (3)	C1—N2—C4—C3	1.1 (4)
N3—C8—C7—S1	−147.93 (19)	C1—N2—C4—C5	−179.3 (3)
C1—S1—C7—C8	66.6 (2)	C2—C3—C4—N2	−0.3 (5)
C8—N3—C9—C14	−164.3 (3)	C2—C3—C4—C5	−179.9 (4)
C8—N3—C9—C10	15.6 (4)	C9—C14—C13—C12	−0.8 (5)
N1—C1—N2—C4	−0.6 (4)	C14—C13—C12—C11	0.6 (5)
S1—C1—N2—C4	179.7 (2)	C13—C12—C11—C10	0.1 (6)
C1—N1—C2—C3	1.5 (4)	C9—C10—C11—C12	−0.8 (5)
C1—N1—C2—C6	−178.3 (3)		
